# Feeling a deep sense of loneliness: Chinese late‐life immigrants in New Zealand

**DOI:** 10.1111/ajag.13108

**Published:** 2022-07-05

**Authors:** Ivy Yan Zhao, Eleanor Holroyd, Valerie A. Wright‐St Clair, Shan Shan Wang, Nick Garrett, Stephen Neville

**Affiliations:** ^1^ WHO Collaborating Centre for Community Health Services The Hong Kong Polytechnic University Hong Kong China; ^2^ AUT Centre for Active Ageing, School of Clinical Sciences Auckland University of Technology Auckland New Zealand; ^3^ School of Clinical Sciences Auckland University of Technology Auckland New Zealand; ^4^ Department of Biostatistics and Epidemiology Auckland University of Technology Auckland New Zealand

**Keywords:** Chinese, immigrants, late‐life, loneliness, social isolation

## Abstract

**Objectives:**

To explore Chinese late‐life immigrants' perceptions of loneliness and social isolation.

**Methods:**

A qualitative descriptive methodology underpinned this study. In‐depth individual interviews were conducted in Mandarin with purposively recruited participants. The twenty‐three participants in the study had all emigrated from China, were 65–80 years old on arrival and had lived in New Zealand for between 2.5 and 16 years. An inductive thematic analytic process was undertaken. The COREQ checklist was followed to ensure study rigour.

**Results:**

Three themes, ‘high value placed on meeting family obligations’, ‘feeling a deep sense of imbalanced intergenerational reciprocity’ and ‘moving away from filial expectations’, were identified. Confucianist values of ‘women's domestic duty of caring for grandchildren’, ‘filial piety’, and ‘saving face’ to be accepted and respected by others negatively attributed to participants' understandings and experiences of loneliness. To plan for increasing frailty and to avoid family conflict while ameliorating potential loneliness, some participants reluctantly discarded prior customary filial piety expectations in favour of formal aged care options.

**Conclusions:**

Participants' profound sense of loneliness was seen to be attributed to their deeply rooted cultural values and backgrounds from having lived for a significant period of time in China. Loneliness occurred as a result of the resettlement process in later life. These experiences highlight the importance of using cultural framing that takes into account beliefs and adaptations to host societies anticipated during the process of late‐life immigration.

**Policy Impact**

For Chinese late‐life immigrants, experiences of loneliness were further complicated and influenced by the process of immigration and the loss of prior cultural and social capital. A person's cultural background should be taken into account by policy makers when planning community residential care, housing options, and health and social care services.

**Practice Impact**

This paper explores how Chinese late‐life immigrants experience loneliness and a reconfiguration of their old age care expectations. It builds on current knowledge by supplementing existing cultural theory and empirical findings for service providers to understand and address loneliness through a culturally responsive service for both Chinese and other ethnic immigrants.

## 1 INTRODUCTION

In 1987, the Immigration Act of the New Zealand government abolished the traditional immigrant selection criteria based on origin‐country preference, and allowed more Chinese immigrants to live permanently here.^1^ Consequently, many people identifying as Chinese chose New Zealand as their home.^1^, ^2^ Late‐life immigration among older people for the purposes of family reunification is a growing phenomenon in Oceania.^2^ In New Zealand, between 1987 and 2017, there was an immigration wave of Chinese adults of working age,^3^ which led to a large number of older dependent parents immigrating to be reunited with their adult children under the Immigration New Zealand's Parent Category Family Reunion Scheme.^4^ This scheme was suspended in 2017 because of an overfull quota.^3^

Older adults are prone to experiencing loneliness due to a deterioration in health status and other age‐related losses, preventing them from participating in meaningful social activities.^5^ Those who have moved to a new country are at risk of loneliness due to the immigration process and having to adapt to a new culture.^6^ Moreover, loneliness is commonly reported by Chinese late‐life immigrants when residing in host countries.^7^ In addition, late‐life immigrants from China, India, and Korea identified negative resettlement experiences and loneliness in New Zealand.^8^ However, many studies on loneliness do not distinguish late‐life immigrants from older adults who immigrated prior to reaching older adulthood.^8^ Unpacking this distinction is critical to providing accurate health and well‐being data to service providers and policy makers that best serve late‐life immigrants. Chinese adults who immigrated in later life are reported as experiencing difficulty with English as a second language, as well as the emotional effects of changes in filial relationships, culture shock, and social isolation when compared with those who immigrated to New Zealand at a younger age.^9^ Many Chinese, Indian and Filipino adult children of older immigrants living in New Zealand have moved away from the traditional filial expectations and turned to formal aged care services for their parents.^10^ These issues are potential risks for loneliness of Chinese late‐life immigrants as previously reported in Australia and Canada.^7^ Further evidence is needed to understand the nuanced experiences of loneliness and the potential links between the disruption of traditional core values associated with filial piety and the experience of loneliness.

In New Zealand, the Health of Older Persons' Strategy^11^ identified loneliness and social isolation as significant health and well‐being issues. The Better Later Life‐He Oranga Kaumatua 2019–2034 strategy,^12^ published by the Office for Seniors, has prioritised addressing loneliness as a key action by promoting opportunities for social engagement and active participation in community activities. Although older adults' loneliness and social isolation is a priority health and well‐being focus for the New Zealand government,^12^ there has been limited research undertaken to understand the Chinese late‐life immigrants' experiences of loneliness in the New Zealand context. There is also scant evidence about the sociocultural adaptations that Chinese late‐life immigrants' experience and the impact this has on loneliness within host countries.^13^ Therefore, the aim of this study was to explore the Chinese late‐life immigrants' perceptions of loneliness and social isolation in New Zealand.

## 2 METHODS

### 2.1 Design

Following the general principles of naturalistic inquiry,^14^ a qualitative descriptive methodology was selected. Using this methodology enabled participants to describe their perceptions of loneliness and/or social isolation and ensured that the researchers presented an accurate representation of participants' accounts.^15^ Furthermore, individual face‐to‐face interviews were deemed more appropriate than focus groups as it is known that some Chinese late‐life immigrants may feel uncomfortable speaking about their experiences of loneliness and issues related to family in a group environment.^16^ We utilised the Consolidated Criteria for Reporting Qualitative Research (COREQ) checklist^17^ to report the study.

### 2.2 Participants

Twenty‐three Chinese late‐life immigrants participated in the study. A purposive recruitment strategy was employed. The inclusion criteria for this study were that participants (1) were older adults from mainland China, Hong Kong, Macau, or Taiwan; (2) were able to communicate in Chinese (Mandarin/Cantonese); (3) were aged 65 years or above upon arrival in New Zealand; (4) had lived in New Zealand for 1 year or more post‐immigration (5) were able to understand the purpose of the study; and (6) could recall their experiences of immigration. Those who immigrated before 65 years of age were excluded.

### 2.3 Ethics

The Auckland University of Technology Human Ethics Committee granted ethical approval for the research [17/55]. An information sheet was provided to participants and written consent gained before commencing data collection. Participant numbers were assigned and used to ensure anonymity of the participants when reporting the findings.

### 2.4 Recruitment and data collection

To recruit eligible participants for the study, flyers (in Chinese) were distributed in places where Chinese late‐life immigrants frequented, for example, community centres, churches, and libraries. In addition, the first author arranged several information sessions to answer people's questions related to the research project. Those who were interested in participating in the study were directed to make contact with the first author. The first author contacted those who were interested and checked their eligibility to participate. Twenty‐seven participants initially responded to the flyers, with 23 participants meeting the inclusion criteria and providing informed consent. Four participants did not meet the inclusion criteria because they had immigrated to New Zealand before the age of 65 years. The 23 included participants were aged between 65 and 80 years on arrival, and had lived in New Zealand for between 2.5 and 16 years. All participants were or had been married, and 13 were currently living with their spouse. Approximately 50% of respondents identified that they were currently living with their adult children. All were retired and 82.6% were receiving New Zealand Superannuation or other New Zealand social welfare allowances for housing, health, or travel expenses. New Zealand Superannuation is the government‐funded pension paid to New Zealand residents aged 65 years or over. The socio‐demographic characteristics are presented in Table 1.

**TABLE 1:** Socio‐demographic characteristics of participants

Attribute and category	No.
*Age*	
65–69 years	2
70–79 years	9
80+ years	12
*Gender*	
Male	7
Female	16
*Age on arrival in NZ*	
65–69 years	17
70–79 years	5
80+ years	1
*Years in NZ*	
2.5–5 years	4
5–10 years	6
10 or above	13
*Marital status*	
Married divorced	1
Living as married	13
Widowed	9
*Education*	
Primary school only	4
Secondary school only	7
Tertiary	12
*English ability*	
Fairly well	6
Poorly/not at all	17
*Religion*	
Yes^*^	6
No	17
*Number of visits to China the past year*	17
0	6
1	
*Living with children*	
Yes	11
No	12
*Number of children*	
1	8
2+	15
*Employment*	
Retired	23
*Sources of income*	
Superannuation/other NZ allowances	19
Retirement allowances from China	4

^*^ Four Christians and two Buddhists.

A semi‐structured interview guide was developed from themes derived from undertaking a review of the literature^8^, ^13^, ^18^ (Table 2). Pilot interviews were conducted with three Chinese late‐life immigrants before undertaking data collection. The pilot interview data highlighted the need for modifications to the interview questions, which was undertaken accordingly to ensure we captured a rich and in‐depth understanding of the phenomenon of interest. All face‐to‐face interviews were conducted in Mandarin by the first author, who is a female Chinese national and fully bilingual. Interviews were approximately of 1 h duration at a private location chosen by the participant and these interviews predominantly occurred in their home. The interviews were digitally recorded. After interviewing 23 participants, the research team was satisfied that thematic saturation^19^ was reached and as such, recruitment ceased. Interviews were concurrently transcribed verbatim in Chinese by the first author and independently checked by another Chinese author (author four). Transcripts were returned to participants to seek verification of the content. Changes were made to ensure the accuracy of source data.

**TABLE 2:** Interview semi‐structured guide

Example of questions
Tell me your story about how you immigrated and lived in New Zealand? What is it like to be Chinese and living in New Zealand? Do you think you have a good quality of life? Why/why not? Describe who you socially engage with on a regular basis? Tell me about the social groups you are involved with? Describe the health and social service agencies you engage with? What does being lonely mean to you as an older person? Are there times in your life when you feel lonely? If so, tell me about them. Tell me about the challenges you experience as an older Chinese immigrant? How do you deal with those challenges and who do you turn to for support? What are some of the things you think are important to reduce loneliness in older Chinese immigrants?

### 2.5 Data analysis

Braun and Clarke's^20^ reflexive thematic analysis was deployed and a data‐driven and inductive thematic analytic process was undertaken. The reflexive thematic analysis emphasises the importance of the researchers' role in knowledge production.^20^ Themes were generated at the intersection of the researchers' reflexive engagement with their theoretical knowledge, analytic resources and skills, interpretation, and the data themselves.^20^ All members of the research team participated in the data analytic process. Data analysis started with transcription of data, followed by a process of reading and rereading the transcripts to identify key features of the data. These key features became our initial codes which were further refined to form themes. The technical process of analysis was undertaken independently by the researchers (author one and author four), owing to the importance of capturing the inherent nuances within the Chinese language and preserving the original meaning of the words used by participants. This approach of post‐analysis translation has shown benefits in preserving ‘explicit and implicit meanings embedded in a language, as well as culturally specific expressions and concepts’.^21^ All the codes and preliminary themes were entered into a spreadsheet and translated into English by the first author and checked by author four. All members of the research team reviewed the translated data and consensus was reached on what became the final themes. An audit of 15% of codes was undertaken by the study team, and a constant comparative method was used to triangulate the data.

## 3 RESULTS

Three themes were generated as a result of the data analytic process and are represented as ‘High value placed on meeting family obligations’, ‘Feeling a deep sense of imbalanced intergenerational reciprocity’, and ‘Moving away from filial expectations’.

### 3.1 High value placed on meeting family obligations

All participants had lived the vast majority of their lives in China and significantly valued meeting their family obligations. Participant 2 described that:

In Chinese traditional culture, my personal spiritual needs were less important than my family. I had no choice. Immigrating to New Zealand to support my adult son is my responsibility and obligation.

Participants who had moved to be with their adult children and grandchildren as older adults, felt obliged to support their offspring by providing childcare, and domestic help. Participant 2 further commented:

I prefer to stay in my hometown [in China] with my big family and friends. But my son had a difficult life in New Zealand. My wife and I must fulfil our grandparental obligations. Nobody would help him, only his parents. It was the only reason for us to move to this country.

While these older parents provided support to their adult children and grandchildren, they also expected that their adult children would in turn help them. The following quote draws on China's prior one‐child policy and ingrained cultural beliefs about filial piety to explain a decision to immigrate at an older age:

I have only one son due to the ‘one‐child policy' in mainland China. He is the only one I can rely on. I had to immigrate to New Zealand because I need his care and support. I could not live in China without any guarantee [of support] in my old age.(Participant 6)

In addition to late‐life care arrangements, participants anticipated they would receive emotional and spiritual support from their adult children. Therefore, immigrating for family reunification reasons, followed by a desire for their adult children's ongoing companionship and being close to their immediate family, were identified as important. One participant highlighted:

My partner has passed away. I felt very lonely after I lost my partner. I was hoping my daughter would spend more time with me.(Participant 15)

### 3.2 Feeling a deep sense of imbalanced intergenerational reciprocity

All participants expressed they felt there would be a balanced exchange of intergenerational reciprocity. Family disagreements in relation to Chinese reciprocal expectations impacted on family dynamics resulting in conflict and disharmonious co‐residential living arrangements, leading to feelings of loneliness. Participant 21 identifies:

My deep feeling of loneliness came from being disappointed with my daughter. Intergenerational disagreements and arguments intensified when I educated my granddaughter through my [traditional] approaches which were considered outdated by my daughter and granddaughter. I felt lonely when I was not understood by them.

The intergenerational conflicts identified in the excerpt above highlight generational differences in attitudes towards child‐rearing practices. Feelings of loneliness, in part, arose from a perceived inability of their adult children to meet their cultural obligations of filial piety.

Participant 16’s quote depicts the tensions and conflicts that existed between a mother and daughter. The participant felt her loneliness stemmed from reduced communication and contact with her daughter:

My daughter and I had the most intense arguments when we first lived together. It reached the point where I did not know how to communicate with her. Both of us have our own distinct personality. She felt hurt sometimes because I did not show appreciation for her filial piety. I had to wander outside alone every day to avoid arguing with her. Sometimes when I could not get on the bus, I would have to sit in the rain, I had tears running down [my face]. I cannot speak any English. I felt lonely since I did not have too many places to go or activities to do. I felt my life was so hard by being old.

Participants' stories also showed they felt burdened by their adult children's expectations for them to contribute to family housework:

I felt upset when I was expected to prepare food for the whole family as a 90‐year‐old man. It made me feel unhappy. The loneliness came together with an unhappy mood.(Participant 4)

Expected involvement in housework and caring for grandchildren, in turn, minimised opportunities for social contact outside the home and further contributed to feelings of loneliness and isolation. Participant 2 likened it to being in a ‘prison’. *Hua di wei lao* indicates that these older Chinese parents felt they were too burdened with domestic house chores to participate in any social activities.

My wife and I had sacrificed seven years of our lives to look after three grandchildren. I had no opportunity [time and freedom] to visit China. I did not have much time to go out to [participate in] community activities in Auckland. My life could be described by a Chinese idiom as ‘hua di wei lao’.(Participant 2)

In addition, participants in this study frequently felt powerless to choose freely how they spent their time and where they could live. Some participants revealed their feelings of being underappreciated by, and subservient to, younger family members. Such experiences contributed to their feelings of imbalanced reciprocity:

I felt very upset and subordinated when living together with my daughter's family in New Zealand. By quoting a Chinese idiom, it refers to living under others' fence (Ji Ren Li Xia). For instance, I was unwilling to get up early every morning to cook breakfast for them. My friends and I usually avoided visiting each other if our adult children were at home. We felt [it was] inconvenient [to chat in front of our adult children] and uncomfortable [for us] when they were there. This makes me feel lonely.(Participant 11)

The Chinese idiom *Ji ren li xia* shows that parents in the current study felt they were not valued and frequently overlooked by their children. A male participant was on the verge of tears when sharing his story and experiences of being powerless to manage his own finances independently:

I really admired local New Zealanders who could afford to live in a retirement village. My children keep all my money.(Participant 13)

Moreover, another participant identified the financial demands placed on her by her daughter:

She [the daughter] keeps our superannuation but still asks us to pay her a high rent. A deep feeling of cold surrounds my heart.(Participant 5)

Another participant identified his feelings of loneliness were exacerbated when he felt excluded from significant and meaningful family activities:

I often felt rejected by my son‐in‐law. They always travelled together when going on a holiday but left me behind and alone at home. They used to include me, but now they said the car was full, which is not true. I frequently eat alone in my room. I am very lonely because I don't feel like I belong to this family, my only family.(Participant 4)

Above all, the perceived imbalances in familial reciprocity were seen to undermine traditional Chinese family obligations and relationships, as well as negatively impacting upon these Chinese late‐life immigrants' sense of self‐worth and well‐being. They shared experiences of being disappointed, burdened, subordinated, isolated, and excluded. These experiences further contributed to deep feelings of loneliness.

### 3.3 Moving away from filial expectations

In an attempt to avoid family conflict and being a burden on their adult children, some Chinese late‐life immigrants began to shift their original values of expecting filial piety to that of becoming more independent. Participants' statements showed that some were actively planning ways to no longer rely on their adult children. As participant 23 said:

I believe that loneliness comes from an uncertainty about the future. It is very important to establish an effective social support system with others like us while we are still able.

Accordingly, a number of testimonies illustrated participants' intentions to become less dependent:

My son has moved back to China for a job opportunity but I am going to stay here by myself. I can manage on my New Zealand superannuation rather than expecting financial support from my children.(Participant 20)

Some participants suggested that their expectations of having to live with and seek support from their adult children might change to relying more on social services, for example:

I think social workers could provide practical assistance to older people, especially those of us living alone and feeling lonely.(Participant 6)

Most participants identified a reluctance to continue to go on living with their adult children, with some planning to move to a formal aged care setting in the future. This identifies a shift in expectations and cultural values related to raising a child to care for them in old age. Some participants also sought support from other Chinese immigrants. To reduce feelings of loneliness, some considered moving to locations where more older Chinese people lived, or to where Chinese businesses/markets were clustered as a form of social support. As Participant 9 explains:

Now we want to move to a more convenient place, with good transportation. A place where other Chinese people live. So if we are not able to cook for ourselves or we are sick in the future, we can buy some food from those Chinese restaurants within walking distance or get traditional medicine to make us better.

## 4 DISCUSSION

As New Zealand residents, this cohort of Chinese late‐life immigrants expressed a deep sense of loneliness which manifested in a number of ways. These feelings were attributed to the contrast between their experiences of growing up and living in China where they had been deeply embedded in their traditional cultural beliefs and values and that of their new lives in New Zealand. Our study findings explored Chinese late‐life immigrants' unrevealed loneliness amidst the traditional core value of filial piety, and identified the relationship between loneliness and a deep sense of imbalanced intergenerational reciprocity. Overall, the present study findings affirm and extend conceptions of Chinese late‐life immigrants' loneliness^22^ in two aspects.

Firstly, the alienation experienced in New Zealand, and language barriers they faced exacerbated participants' loneliness and social isolation. Participants felt burdened by being made to undertake housework and having to look after grandchildren, which contributed to feelings of loneliness due to an inability to participate in social activities. The findings of this study are consistent with reports from the United States, Australia, and Canada, where Chinese late‐life immigrants' social networks were considerably restricted, and resulted in increased experiences of loneliness.^23^, ^24^

Secondly, some participants identified being excluded from family activities by younger generations. They commonly related it to an experience of being ‘disowned’ by their adult children. These experiences triggered feelings of loneliness and were further exacerbated when culturally located filial expectations were also not met. These experiences are consistent with the Chinese definition of loneliness: older without a child.^25^ In Australia, Lin^26^ also reported that feelings of loneliness intensified when younger generations of Chinese immigrants did not have enough time to spend with their older parents. However, adult children from immigrant families may perceive and practice traditional filial piety differently from their older parents. According to Montayre's study,^10^ some adult children from immigrant families in New Zealand preferred to modify their ways of providing aged care to their parents while upholding the core values and cultural familial exceptions. Some of them shifted their own aged care expectations from traditional filial values to formal caring services. The mismatch in filial values and practices between immigrant adult children and their older parents linking to Chinese late‐life immigrants' sense of imbalanced intergenerational reciprocity resulted in further perceptions of loneliness.

In addition, some participants found themselves living alone in the host country which was further disappointing their expectations of filial piety. A participant raised an issue that after immigrating, her adult son moved back to China for job opportunities and left her behind in New Zealand. Although the participant expressed that she was able to live independently by relying on her New Zealand Superannuation without her adult son's support, several social service stakeholders in New Zealand have raised concerns on this issue due to the fast‐growing number of older Chinese. Being left behind in a foreign country further exacerbates loneliness which is expressed as feelings of helplessness and being abandoned or overlooked by their families.^27^ This finding parallels with research on Chinese late‐life immigrants' loneliness in both Australia and Canada^23^ that living alone and loneliness were potential risk factors for older people to decrease their physical and psychological health and well‐being. For those people, the COVID‐19 pandemic brought a big challenge to their lives in a host country. Studies in Belgium and Netherlands reveal that social isolation and financial insecurity during the pandemic have led to increased loneliness among older Chinese immigrants.^28^

Understandings and perceptions of loneliness ultimately determine and influence the way Chinese late‐life immigrants respond to and address feelings of loneliness. As previously identified, to overcome loneliness, some participants in this study shifted their care expectations from drawing on filial piety expectations of being cared for by family, to that of relying on formal health and social services. A similar finding is reported by Montayre et al.^18^ with older Filipino immigrants in New Zealand. Consequently, service providers must develop cultural awareness about ageing as a member of an immigrant group and be prepared to deliver culturally relevant healthcare and social services. Furthermore, Arthur Kleinman's Social Suffering Theory^29^ and Wright‐St Clair's research findings^8^ contend that the cultural stigmatisation of loneliness could impose barriers that inhibit Chinese immigrants from seeking assistance or peer support. According to the Social Suffering Theory,^29^ Kleinman suggests that cultural and social forces can influence an individual's psychological health and well‐being, and the suffering usually extends to their family and social network. In Chinese communities, loneliness can be socially stigmatised as an inability to develop interpersonal relationships (*guan xi*), which detrimentally challenges people's capability and impacts the person's face (*mian zi*).^27^ Chinese late‐life immigrants may fear a loss of face if they went to see a doctor or health professional to talk about feeling lonely.^30^ Therefore, it is important for service providers to be aware that Chinese late‐life immigrants may disguise the reality of their current family and living circumstances, as well as any feelings of loneliness and/or abandonment if they perceive they might be stigmatised.

All participants originated from mainland China. Chinese late‐life immigrants from other regions (Hong Kong, Macau, and Taiwan) were considered for recruitment, but they did not meet the inclusion criteria. Chinese late‐life immigrants from other countries and regions and late‐life immigrants from other ethnic groups may have different understandings and experiences of loneliness due to their diverse backgrounds and culturally specific contexts. We acknowledge that the presence of their ‘voices’ could add value to future research. A descriptive‐analytic approach was adopted in this study to stay close to the data. However, the richness of the data may remain underexplored.

## 5 CONCLUSIONS

Loneliness was seen to negatively impact on Chinese late‐life immigrants' well‐being; however, their perception of what it is to be lonely is often poorly understood in the wider community. In addition to the widely recognised social and emotional factors attributed to loneliness, findings from this study suggest that Chinese late‐life immigrants' feelings of loneliness were culturally located. Their traditional expectations of filial piety were seen as a disruption and resulted in a need for new configurations to accommodate their future care needs. Findings from this study highlight that attention to cultural competency is an essential sensitising lens for researching or delivering initiatives to address loneliness in late‐life immigrant groups.

## CONFLICTS OF INTEREST

Professor Stephen Neville is a member of the Editorial Board of the Australasian Journal on Ageing. No other conflicts of interest are declared.

## Data Availability

Data available on request due to privacy/ethical restrictions.
